# Continued investigator engagement: Reasons principal investigators conduct multiple FDA-regulated drug trials

**DOI:** 10.1016/j.conctc.2019.100502

**Published:** 2019-11-23

**Authors:** Carrie B. Dombeck, Terri Hinkley, Christopher B. Fordyce, Katelyn Blanchard, Matthew T. Roe, Amy Corneli

**Affiliations:** a Clinical Trials Transformation Initiative, Durham, NC, USA; b Department of Population Health Sciences, Duke University, Durham, NC, USA; c Academy of Medical-Surgical Nurses, Sewell, NJ, USA; d Duke Clinical Research Institute, Durham, NC, USA; e Division of Cardiology, University of British Columbia, Vancouver, British Columbia, Canada

**Keywords:** FDA, Drug trials, Investigator turnover, Principal investigator, Physician-investigator, Clinical investigator

## Abstract

**Background/Aims:**

Numerous reasons have been identified for why U.S.-based principal investigators choose to not continue participating in FDA-regulated trials. However, unexplored are reasons why a substantial number of principal investigators, facing the same challenges, remain engaged in clinical research. This study aimed to both describe barriers and identify factors that contribute to active investigators’ success in conducting multiple FDA-regulated trials.

**Methods:**

We conducted qualitative in-depth interviews (IDIs) with “active” multi-trial investigators. Interviews focused on investigators’ experiences with FDA-regulated drug trials, challenges faced, and factors contributing to success. Investigators also reflected on previously identified barriers and shared advice for new investigators. Narratives were analyzed using applied thematic analysis.

**Results:**

We interviewed 23 experienced investigators, representing a variety of backgrounds. Most reported that demonstrated ability to conduct a trial led to being approached again by sponsors. Investigators cited infrastructure, staff support, advance planning, and personal qualities as key factors in successfully conducting multiple trials. Nearly all cited difficulties related to trial finances. Three-quarters pointed to challenges with patient recruitment; others described challenges related to data and safety reporting and to the time that trial implementation takes away from other activities. Aspiring investigators were advised to engage in research-specific training and seek out mentorship opportunities.

**Conclusion:**

Investigators in our sample faced many of the same challenges identified in previous research, yet they had evolved strategies to overcome them. The amount and type of support to which investigators have access may represent a crucial difference between “active” investigators and principal investigators who leave FDA-regulated trials.

## 1 Introduction

Research suggests that the number of one-time principal investigators of drug trials regulated by The U.S. Food and Drug Administration is on the rise [1]. Attrition from clinical trials is not limited to first-time investigators, as scholars note that challenges facing the clinical research enterprise are also causing some veteran investigators to leave research [2]. Publicly available data in the Bioresearch Monitoring Information System (BMIS) database show that annually, approximately 40% of unique investigators choose not to participate in another FDA-regulated trial [3]. This exodus of experienced clinical investigators, as well as the time and resources required to initiate new investigators into the clinical trial process, are believed to be linked to inefficiency, instability, and increased costs associated with conducting clinical trials [1].

Principal investigators contend with many challenges related to trial implementation and conduct, including site infrastructure issues [2], lack of institutional support [4,5], competing priorities [4], difficulties in making research financially viable for investigators [6], and long timelines and administrative burden [7]. In our own previous research [8], we identified reasons why U.S.-based investigators who conducted only a single FDA-regulated drug trial (“one-and-done” investigators) chose not to continue with clinical research as a principal investigator. Our findings provide empirical support to many issues described in the literature, such as balancing other clinical and work obligations with the time requirements necessary for conducting FDA-regulated clinical trials [8].

One aspect of investigator turnover that remains largely unexplored is why, in the face of numerous obstacles, a substantial number of principal investigators remain engaged in clinical research, in some cases conducting hundreds of trials over the course of their careers. In this manuscript, we report on findings from interviews with currently active investigators who have conducted multiple FDA-regulated drug trials. The objective of this study was to identify reasons for the active investigators’ success in conducting multiple trials, to describe challenges experienced or avoided by these investigators, and to characterize strategies used by investigators to manage and/or prevent these challenges.

## 2 Methods

The Clinical Trials Transformation Initiative (CTTI) (http://www.ctti-clinicaltrials.org) is a public-private partnership established by FDA and Duke University that seeks to identify and drive adoption of practices that increase the quality and efficiency of clinical trials. As part of a larger CTTI effort to address the issue of investigator turnover, we previously administered an online survey [8] to U.S.-based principal investigators who were documented in the BMIS database as having conducted only one FDA-regulated drug trial (referred to as “one-and-done” investigators). This survey identified the reasons why investigators chose to conduct only one trial and focused on obtaining an understanding of the barriers to trial participation that principal investigators face.

Building on this previous work, the present study focused on investigators who remain actively engaged in conducting FDA-regulated drug trials, to explore how and why they were able to conduct multiple trials, whether they have experienced any of the same challenges identified by the “one-and-done” investigators as barriers to conducting further trials, and if so, how they were able to manage and overcome these challenges.

### 2.1 Study design

We conducted a qualitative descriptive study [9,10], using in-depth interviews (IDIs).

### 2.2 Eligibility, participant selection, and recruitment

Investigators were purposefully selected [11] and were eligible to participate if they 1) were an “active” investigator, as defined as an investigator who served as the principal investigator in a minimum of four distinct FDA-regulated drug trials (i.e., with separate FDA Forms 1572) in the two years prior to June 1, 2016; and 2) had been involved in multiple aspects of trial conduct (e.g., protocol development and study implementation).

Participants were recruited via announcements on two investigator listservs: the Society for Clinical Research Sites and the Association of Clinical Research Professionals. Invitation emails were also sent to investigators listed in two academic investigator databases. Interviews were conducted between July 20 and October 12, 2016.

### 2.3 Data collection

**Fig. 1:**
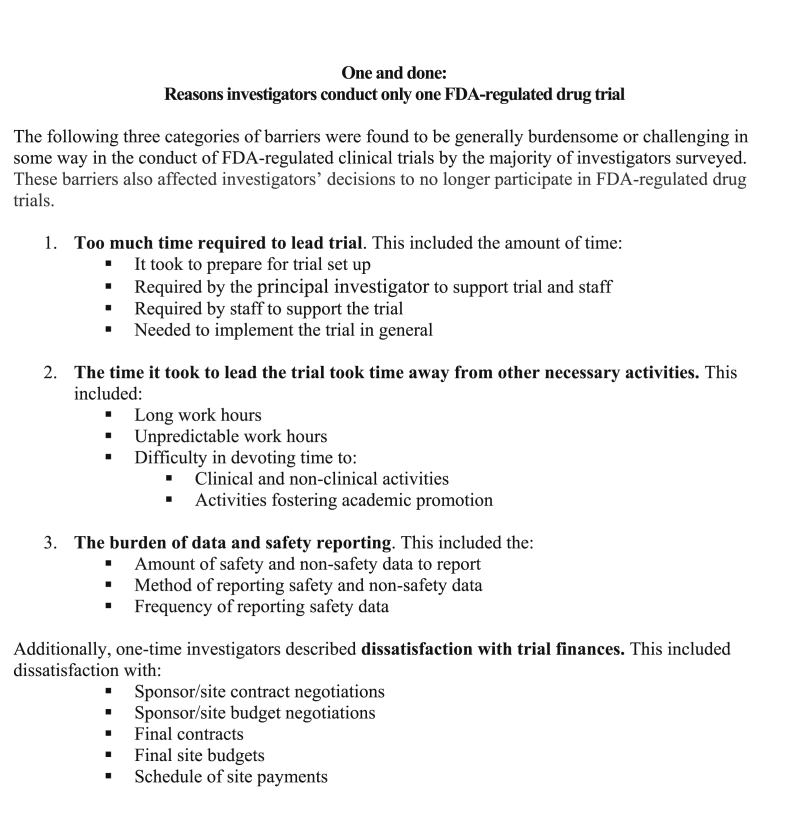
Findings from Corneli et al., 2017 reviewed by investigators during the interviews.

All interviews were conducted on the telephone by one of two interviewers. Demographic information was collected prior to the interview. During the first part of the interview, investigators described how they initially became involved in FDA-regulated drug trials and were asked to discuss the factors that they believe enabled them to successfully conduct multiple trials. Next, investigators were asked to describe any challenges they had faced in conducting FDA-regulated drug trials and how they had dealt with these challenges. Prior to the interview, investigators were provided with the list of barriers identified in our previous survey with “one-and-done” investigators (Fig. 1); the “active” investigators were asked to reflect on these barriers during the interview. Specifically, they were asked to describe 1) if they had experienced these types of challenges and if so, how they managed or coped with them or 2) if they did not experience such challenges, reasons why they believed they had been able to avoid them. Lastly, investigators were asked to share advice for new investigators on how to become involved in FDA-regulated drug trials. All interviews were digitally audio-recorded, with participants’ permission.

### 2.4 Data analysis

Interviews were professionally transcribed verbatim following a standardized transcription protocol [12]. Participants' narratives were analyzed using applied thematic analysis [13]. Using NVivo 11 (QSR International Pty Ltd., 2010), three analysts first applied deductive structural codes to segment participants' dialogue into conceptual categories, based on the topics explored in the interview. Inter-coder reliability assessments (ICRs) were conducted on 17% of the transcripts, and discrepancies in code application were resolved through discussion. Following the ICRs, transcripts were re-coded as needed, and edits were made to the codebook to aid in code application to future transcripts. Next, data reports from similar structural codes were grouped together based on their relationship to the study's objectives. Four analysts inductively identified and applied thematic codes to the narratives across the structural coding reports for each objective, which allowed for identification of issues salient to participants. Analysts then produced summary reports that described the major themes and sub-themes.

This study was granted a determination of exempt status by the Duke University Health System Institutional Review Board. All participants were provided with an informational sheet prior to study participation that explained the study in detail, including its purpose, risks, and benefits.

## 3 Results

### 3.1 Study population

We interviewed 23 investigators: 7 from academic sites, 8 from community sites, and 8 from dedicated research sites (Table 1). The median age was 58 (range: 44 to 72), approximately three-fourths (n = 18) were male, and most were white (n = 21). Nearly all were MDs; two were DOs. Investigators represented 11 specialties and had been employed at their current institution between 2 and 41 years (median: n = 10). Investigators had served as the principal investigator of an FDA-regulated drug trial for 7 to 43 years (median: n = 17). Since their first trial, investigators reported conducting a total of 10 to 500 (median: n = 50) FDA-regulated drug trials as the principal investigator, with 5 to 100 of these trials (median: n = 15) having been conducted within the past two years. All investigators had been sponsored by industry, and just over half (n = 13) reported having U.S. government sponsors. All had conducted Phase 3 FDA-regulated drug trials, and many had also conducted Phase 1 and 2 trials. All investigators reported having at least some sites located in the U.S.

**Table 1:** Investigator demographics and experience with FDA-regulated drug trials, by investigator type.

Variable	Investigator Affiliation			
	Academic Institution n = 7	Community-Based (Private Practice or Hospital) n = 8	Dedicated Research Site n = 8	Total n = 23
**Gender**				
Male	4	7	7	18
Female	3	1	1	5
**Age**				
35-44	0	1	0	1
45-54	2	3	2	7
55-64	2	3	5	10
65-74	3	1	1	5
**Race**				
Asian	0	0	1	1
Black or African American	0	1	0	1
White	7	7	7	21
**Ethnicity**				
Hispanic or Latino	1	0	2	3
Not Hispanic or Latino	6	8	5	19
Prefer Not to Respond	0	0	1	1
**Terminal Degree**				
MD	7	6	7	20
MD/PhD	0	0	1	1
DO	0	2	0	2
**Specialty**				
Allergy and Asthma	0	1	0	1
Cardiology	1	1	1	3
Endocrinology	1	0	1	2
Melanoma and Sarcoma	1	0	0	1
Neurology	0	0	1	1
OB/Gyn	0	1	1	2
Ophthalmology	0	1	0	1
Family and Internal Medicine	0	2	3	5
Pediatric Medicine	4	0	1	5
Pulmonary	0	1	0	1
Uro-gynecology	0	1	0	1
**Years employed at current institution**				
Median (range)	20 (2–41)	18 (6–32)	10 (2–19)	16 (2–41)
**Number of years conducting FDA-regulated drug trials as a PI**				
Median (range)	20 (9–25)	14 (8–43)	17 (7–33)	17 (7–43)
**Total number of FDA-regulated drug trials conducted as the PI, lifetime**				
Median (range)	20 (10–100)	50 (10–200)	138 (38–500)	50 (10–500)
**Total number of FDA-regulated drug trials conducted as the PI, past 2 years**				
Median (range)	14 (5–20)	9 (5–75)	25 (6–100)	15 (5–100)
**Sponsors of FDA-Regulated Drug Trials**^a^				
Pharmaceutical industry	7	8	8	23
U.S. government	7	2	4	13
Private foundation	2	2	1	5
Non-governmental organization	1	0	0	1
Investigator-initiated and funded	3	1	0	4
Other	0	0	1	1
**Phases of Clinical Research**^a^				
Safety trial (typically Phase I)	4	3	5	12
Proof of concept or dose-ranging trial (typically Phase IIa/b)	5	6	8	19
Pivotal trials for registration (typically Phase III)	7	8	8	23
Post-marketing	4	6	8	18
**Study Locations**^a^				
Inside the US only	4	6	7	17
Outside the US only	0	0	0	0
Inside and Outside the US	3	2	1	6

a Investigators selected all that applied for all previous FDA-regulated clinical trials they conducted as the PI.

Investigators’ first exposure to FDA-regulated drug trials was often either as a fellow working directly with the trial principal investigator, or as a sub-investigator/co-principal investigator of an FDA-regulated trial. For a few, their first time as a principal investigator was their first exposure to FDA-regulated drug trials. Investigators were split on when in their career they first served as the principal investigator of FDA-regulated research, with some reporting that this took place early in their career (i.e., 1 to 3 years after residency), while for others this did not occur until after they had been practicing medicine for 10 or more years.

### 3.2 Accessing opportunities to conduct FDA-regulated drug trials

Most of the investigators interviewed cited direct contact with a sponsor as the means by which they first became principal investigator of an FDA-regulated drug trial. After this initial experience, investigators used various means to remain involved with trials. Most reported being approached again by sponsors about new trial opportunities after they had successfully demonstrated that they had the necessary infrastructure and ability to conduct a trial, noting that investigator reputation matters and “...it's been driven through success.” Many investigators also described learning about trials through connections with clinical trial networks or colleagues, or by reaching out to pharmaceutical companies to inquire about new trial opportunities.

### 3.3 Factors contributing to principal investigator success in conducting multiple FDA-regulated drug trials

All of the active investigators interviewed cited staff support as a key factor behind their success in conducting more than one FDA-regulated drug trial, noting that it is essential for trials to have sufficient and well-trained staff. In this regard, the study coordinator was viewed as the most important member of the research team, but investigators also described the importance of other site personnel, such as administrative, regulatory, and pharmacy. For academic investigators, institutional support and resources were also seen as critically important, particularly in terms of being provided with protected time to devote to research. Further, many investigators noted the importance of their own personal qualities, such as having a good work ethic, to their success in conducting multiple trials. Given that clinical research often involves long hours for limited pay, investigators described that it is important to be personally committed to the work, enjoy doing it, and believe in its importance.

Several other factors relating to successfully conducting multiple trials were also mentioned by investigators, although less frequently; these included having the ability to recruit sufficient participants, being selective with trials, and choosing only quality protocols. Investigators noted that thinking through their ability to recruit the promised number of participants prior to accepting a trial, and declining if they think it will be difficult to reach the target sample size, as well as choosing studies that they think will be successful, are important factors to consider for ensuring that they maintain a reputation for conducting quality research and thus continue to be offered trial opportunities.

### 3.4 Challenges faced and strategies for managing them

Nearly all of the active investigators interviewed identified trial finances as a challenge they had faced. Tight budgets, rendering investigators unable to sufficiently cover the resources needed to conduct a quality trial, were most frequently noted as a difficulty; investigators highlighted an emerging trend of diminishing funding for increasing amounts of work and responsibility. Investigators also expressed frustration with budget negotiations, payment delays, inaccurate budget forecasting, and competition for limited funds. To address issues related to trial finance, successful investigators described strategies along the continuum of study conduct, but largely focused on the importance of pre-planning and realistic assessment of costs as a means of addressing budget shortfalls during the negotiation phase. Investigators also pointed out the importance of being selective and declining or ending trials if the budget was unsuitable (Table 2).

**Table 2:** Common coping mechanisms and strategies used to address trial finance challenges.

**1** **Assess: Go or no-go?** • Conduct a pre-assessment o Review protocol carefully o Determine feasibility of recruiting minimum number of patients • Be selective when accepting trials • Decline trials **2** **Reduce barriers: Know your costs and/or rely on staff to help** • Identify what is needed for operations • Know your own costs • Develop fee schedule • Clarify fees upfront, especially start-up costs • Rely on staff to follow-up on payments not received • Understand budget line items • Follow monthly budget **3** **Communicate** • Negotiate and push back • Have investigator decide on payment terms and schedule • Communicate with sponsor about cutting losses • Wait for sponsor to return at later point to negotiate

Almost all active investigators also mentioned challenges related to the time it takes to lead a trial. They commented that the actual time it takes to complete a trial is often more than anticipated, leading to a need for either more staff time or more staff members than originally budgeted. Yet investigators have found it extremely difficult to renegotiate for additional coverage. Investigators also expressed frustration with start-up delays, which often result from the need to wait for supplies, regulatory approvals, and FDA review. As with trial finance, investigators largely cited coping mechanisms related to the pre-planning phase, noting that this is a critical time for attempting to accurately identify and communicate the amount of time it will take to conduct the trial. Further, investigators noted the importance of establishing a supportive infrastructure, with well-trained staff who can take some of the workload off the principal investigator. As one investigator commented, investigators should “…really have a team behind you to do this. Without that, it's virtually impossible… As physicians, we are way too busy to try to do all of that without the support.” Others commented on the necessity of anticipating inevitable delays and simply accepting the time commitment (Table 3).

**Table 3:** Common coping mechanisms and strategies used to address challenges with the time required to implement the trial.

**1** **Establish a supportive infrastructure** • Staffing o Hire appropriate staff positions and ensure they are talented, a good fit, and well-trained o Have a regulatory employee to help review protocols and contracts o Have a good study coordinator • Systems o Develop and use working systems **2** **Prepare prior to trial implementation** • Use knowledge and experience when reviewing protocols to identify time commitments—or gain experience and rely on it • Anticipate delays to start-up • Do not assign staff to trial until it is up and running • Communicate with sponsor to prevent unanticipated time burdens **3** **Reality check** • Work overtime, weekends, and nights • Accept time commitment • Deal with delays

Three-fourths of investigators identified as an obstacle the difficulty of finding and recruiting patients who met the trial's eligibility criteria. Investigators noted that sponsors' eligibility criteria were often unrealistic or so stringent they affected the investigators' ability to conduct the trial, thus affecting study timelines and budgets. This was highlighted as indicative of an industry-wide trend, in which less clinician and investigator input during protocol development leads to trial designs and eligibility criteria that may not be appropriate for the population, feasible, or scientifically useful. As with the other challenges, strategies related to planning/assessment and communicating with the sponsor were most often noted, such as reviewing draft protocols for feasibility and being upfront about expected recruitment numbers. Investigators also remarked on the importance of using a variety of recruitment strategies (Table 4).

**Table 4:** Common coping mechanisms and strategies used to address recruitment and eligibility challenges.

**1** **Plan appropriately** • Review protocol to assess its feasibility o Use multiple reviewers and perspectives (e.g., investigator and coordinator) o Use experience and knowledge of study population o Review existing patient database • Adjust protocol • Become involved in protocol development • Decline trials **2** **Address potential barriers** • Recruit patients from own private practice • Use a variety of recruitment strategies • Listen to patients: obtain feedback, engage potential participants **3** **Communicate** • Provide feedback on adjusting trial and criteria • Ask sponsor questions about protocol and eligibility criteria • Discuss recruitment and criteria challenges with sponsor • Be upfront with sponsor about expected recruitment numbers • Push back on criteria and medical monitors and sponsors during trial **4** **Get a reality check** • Just deal with it • Keep looking for more patients

Almost two-thirds of active investigators said they found the burden of data and safety reporting to be challenging. The sheer quantity of reporting required was seen as overwhelming and as taking away time and resources from other trial-related duties. While investigators accepted the necessity of reporting AEs and SAEs, in some cases they noted burden due to CROs and sponsors requesting information that was excessive or not relevant to trial safety, or requiring sign-offs from too many people. To mitigate this, investigators again focused on the importance of infrastructure and institutional support, citing the importance of dividing labor among quality staff, and creating standardized procedures to help the process run smoothly.

Regarding other barriers to conducting multiple FDA-regulated drug trials that were presented to investigators during the interview, one-third of investigators agreed with the “one-and-done” investigators that the time trial implementation takes away from other activities is challenging, particularly as relates to obtaining salary coverage. Another one-third noted challenges related to working with regulatory agencies, sponsors, and CROs, while a handful of investigators commented on challenges due to poor-quality protocols. Preparation, communication, being able to prioritize, and having institutional support were all highlighted as helpful strategies for holding these issues at bay.

### 3.5 Recommendations for becoming an active investigator

Investigators who shared advice for new investigators on how to become involved in FDA-regulated drug trials most commonly suggested engaging in research-specific education or training, such as fellowships. Several also advised new investigators to find a mentor or otherwise network with more experienced colleagues, and some recommended serving as a sub-investigator, as a means of gaining trials experience without having full responsibility for the research. Investigators advised to: “…do some trials, do them well, recruit well, do good data, and then that automatically gets the word out there for you…, that you're a good researcher.” Active investigators felt that those just starting out in research should have the same personal qualities that they credited for their own success, including interest in and enjoyment of research and attention to detail. Finally, experienced investigators again stressed the importance of pre-planning, having a full understanding of what is expected from the role before committing, bringing quality staff on board, having institutional support, and engaging in time and financial management.

## 4 Discussion

Our study yields several important considerations for sustaining and growing the participation of productive, experienced investigators in FDA-regulated drug trials. Among our study population, most of the investigators interviewed had experienced many of the same challenges as the one-time investigators surveyed in our previous research [8]. Yet, they had evolved strategies to manage and cope with these barriers. The amount and type of support to which investigators have access among the one-and-done investigators and the active investigators is unknown but may represent a crucial difference between these two populations; having a dedicated, experienced study coordinator and sufficient study personnel was cited by those interviewed both as one of the key elements of their success in conducting multiple trials, and as a strategy for managing a variety of research-related challenges. For academic investigators, having institutional support and commitment was also viewed as critically important, both in terms of provision of resources and infrastructure, and perhaps more significantly, in terms of a supportive institutional research culture that protects time for investigators to engage in research activities.

The importance of advance planning and preparation, prior to trial initiation, was also highlighted multiple times by successful investigators. Pre-planning and engaging in feasibility assessments to identify issues up-front were seen as vitally important to managing challenges associated with budgeting, study timelines, and ability to meet recruitment goals. During the planning stage, investigators also stressed the importance of having good communication with sponsors, in order to set realistic expectations and enable push-back on unsuitable budgets and eligibility criteria. Investigators noted that failure to perform due diligence on a protocol ahead of time could lead to later difficulties with study implementation, potentially resulting in extended timelines and inflated budgets. Given that investigator performance was viewed as an important aspect of successfully conducting multiple trials, spending some time carefully assessing the principal investigator responsibilities inherent in each potential new study could ultimately maximize the investigator's chances of being offered subsequent drug trials.

Our findings also show that intangible characteristics, such as investigators’ personal traits, may contribute to success in conducting multiple trials. The investigators we interviewed cited attention to detail, good time management skills, enjoyment of the research process, and acceptance of the inevitability of difficulties as some of the qualities that helped them in this regard. Successful investigators advised those just beginning their clinical research careers to start small, seeking out mentorship or fellowship opportunities before taking on full responsibility for a trial. Investigators believed that learning the ropes of clinical research in a lower-stakes environment could provide new investigators with plenty of opportunities to gain experience, which could presumably also help them to determine whether they are temperamentally a good fit for the stresses of drug trials; those who are not may be more likely to self-select out of clinical research at an early stage, rather than going on to serve as the principal investigator for subsequent studies.

A strength of our study was that we asked investigators to reflect on the factors that they saw as contributing to their success in conducting more than one FDA-regulated drug trial, as well as specifically asking them to enumerate strategies that they used to cope with or manage obstacles to conducting clinical research. Although some of these factors, such as the research culture of individual institutions, may be outside of investigators' control, we nonetheless identified a number of specific, actionable strategies that motivated researchers can use to mitigate challenges to conducting subsequent trials. We also confirmed findings of specific barriers identified in our own and others’ research; future efforts to reduce investigator attrition could focus on reducing or eliminating the causes of these barriers.

We also note some limitations to our study. Although we obtained a diverse sample of investigators from three different types of institutions—academic, community, and dedicated research sites, some of the investigators’ recommendations may only apply to certain research settings. Additionally, most of the participants were white males; investigators from other demographic groups and a different sample of investigators may have identified other barriers and solutions.

## 5 Conclusions

Our research confirmed and extended previous work among clinical investigators that identified specific barriers to continuing on in clinical research. While the current study population of active investigators of multiple FDA-regulated drug trials likewise found these barriers to be frustrating, they had evolved numerous strategies for managing these challenges and mitigating their effects. Future efforts to address barriers, and implementation of the strategies identified for coping with them, could help reduce attrition of experienced investigators in the future.

## Funding

Funding for this manuscript was made possible, in part, by The U.S. Food and Drug Administration through grant R18FD005292 and cooperative agreement U19FD003800. Views expressed by the authors do not necessarily reflect the official policies of the Department of Health and Human Services, nor does any mention of trade names, commercial practices, or organization imply endorsement by the US Government. Partial funding was also provided by pooled membership fees and in-kind contributions from CTTI's member organizations.
